# Balancing Hole and Electron Conduction in Ambipolar Split-Gate Thin-Film Transistors

**DOI:** 10.1038/s41598-017-04933-w

**Published:** 2017-07-10

**Authors:** Hocheon Yoo, Matteo Ghittorelli, Dong-Kyu Lee, Edsger C. P. Smits, Gerwin H. Gelinck, Hyungju Ahn, Han-Koo Lee, Fabrizio Torricelli, Jae-Joon Kim

**Affiliations:** 1 Department of Creative IT Engineering, Pohang University of Science and Technology (POSTECH), Pohang, 790-784 Korea; 2 Department of Information Engineering University of Brescia, via Branze 38, 25123 Brescia, Italy; 3 Holst Centre, TNO-The Dutch Organization for Applied Scientific Research High Tech Campus 31, 5656 AE Eindhoven, The Netherlands; 4 Pohang Accelerator Laboratory, Pohang University of Science and Technology (POSTECH), Pohang, 790-784 South Korea; 5 Department of Applied Physics, Eindhoven University of Technology, 5600 MB Eindhoven, The Netherlands

## Abstract

Complementary organic electronics is a key enabling technology for the development of new applications including smart ubiquitous sensors, wearable electronics, and healthcare devices. High-performance, high-functionality and reliable complementary circuits require n- and p-type thin-film transistors with balanced characteristics. Recent advancements in ambipolar organic transistors in terms of semiconductor and device engineering demonstrate the great potential of this route but, unfortunately, the actual development of ambipolar organic complementary electronics is currently hampered by the uneven electron (n-type) and hole (p-type) conduction in ambipolar organic transistors. Here we show ambipolar organic thin-film transistors with balanced n-type and p-type operation. By manipulating air exposure and vacuum annealing conditions, we show that well-balanced electron and hole transport properties can be easily obtained. The method is used to control hole and electron conductions in split-gate transistors based on a solution-processed donor-acceptor semiconducting polymer. Complementary logic inverters with balanced charging and discharging characteristics are demonstrated. These findings may open up new opportunities for the rational design of complementary electronics based on ambipolar organic transistors.

## Introduction

Organic electronics have been extensively investigated over the last decade^1–3^ for a wide range of new applications including healthcare^4, 5^, smart sensors^6–8^, wearable devices^9, 10^, etc. The aforementioned applications urgently require complementary technologies where both electron (n-type) and hole (p-type) conducting organic thin-film transistors (OTFTs) are available. In conventional complementary OTFT technologies, the fabrication of p- and n-type transistors requires the separate deposition, patterning and optimization of two different semiconducting materials^11–16^, one for each transistor type^17, 18^. While this may seem to be a simple approach, in practice, the n-type and p-type organic semiconductors need to exhibit comparable electronic properties, requiring ad-hoc process conditions and optimizations^19, 20^. These may result in complex fabrication procedures, which are not desirable for low-cost and mass production.

Ambipolar organic semiconductors, in which both holes and electrons can be injected and transported in a single semiconducting layer, significantly reduce the complexity of the fabrication processes. Depending on the applied voltage, ambipolar OTFTs can operate as a p-n junction, or p-type and n-type transistors^21, 22^. Therefore, ambipolar OTFTs have attracted considerable attention for their application in complementary metal–oxide semiconductor (CMOS) digital integrated circuits^23, 24^ and light-emitting field-effect transistors^25, 26^.

Continuous transitions from the hole-conduction state to the electron-conduction state are typically observed in ambipolar OTFTs. In other words, these transistors do not have large on-off current ratios as they do not have a well-defined off-state region. As a result, ambipolar conduction results in large power consumption in electronic circuits. One of the approaches to overcome this fundamental limitation is to modify the gate dielectric and/or the charge injecting contacts, so that the polarity of the transistor is permanently set^22, 27^. Another approach is to control the polarity of the transistor by using multiple gate electrodes. Recently, multi-gate techniques such as split-gate and tri-gate architectures have been demonstrated as viable and effective approaches to electrostatically select the polarity of ambipolar OTFTs^28–32^. Depending on the voltage bias of the secondary-gate, an ambipolar OTFT can operate as either a unipolar p-type or n-type transistor. However, the balance between hole and electron conduction in ambipolar semiconductors still remains a key requirement for achieving high-performance complementary electronics. The imbalance between hole and electron transport reduces the noise immunity and the DC gain in a logic inverter. Despite recent improvement in terms of charge transport (mobility > 1 cm^2^ V^−1^ s^−1^)^33, 34, 35^ most organic ambipolar semiconductors exhibit a significantly larger hole mobility than electron mobility^36, 37^ or vice versa^34, 35^. In addition, the turn-on voltages of p-type and n-type are typically different in ambipolar OTFTs^38, 39^. Previous research for balancing electron and hole current has focused on designing new materials^40–43^ or devising a new approach to blend^44, 45^ or stack^46, 47^ two materials. While such approaches are very promising, the balance between hole and electron currents may vary depending on the device structure^40, 43^ or manufacturing process^42^. Therefore, there is a great need to more clearly understand and control the p-type and n-type charge transport properties through a comprehensive physical analysis.

Electrical characteristics of OTFTs depend on the environmental conditions (ambient, vacuum or nitrogen atmosphere)^48, 49^. In particular, air exposure has a significant effect on charge transport properties. For example, it has been reported that degradation of electron transport occurs in n-type semiconductors due to interaction with oxygen and/or water^50, 51^. In addition, enhancing p-type hole transport by intentionally allowing interaction with diluted air has been also reported^52^. The degree of interaction with air is even more important in ambipolar OTFTs since both holes and electron transports need to be considered simultaneously. It has been reported that benzodifurandione-based oligo (*p*-phenylene vinylene) (BDOPV) exhibited ambipolar characteristics when fabricated under ambient condition while the same polymer showed unipolar n-type transport under nitrogen atmosphere^34^. This indicates that the amount of air exposure can be a key design parameter to control electron and hole current in an ambipolar OTFT with a careful study of atmospheric effect.

In this work, we show that hole and electron transport can be balanced by controlling the air exposure time and the temperature of the thermal treatment in poly-(diketopyrrolopyrrole-terthiophene) (PDPP3T) ambipolar semiconducting polymer. The analysis is based on electrical measurements, ultraviolet photoelectron spectroscopy (UPS), X-ray photoelectron spectroscopy (XPS), and two-dimensional (2D) numerical simulations. Based on this comprehensive analysis we demonstrate split-gate ambipolar OTFTs with balanced hole and electron characteristics. When the devices are connected in a complementary inverter configuration, larger gain and output swing were achieved compared to the values from inverters with unbalanced n/p characteristics.

## Results

### Experimental observation of impact of the air-exposure on the transistor properties

**Figure 1:**
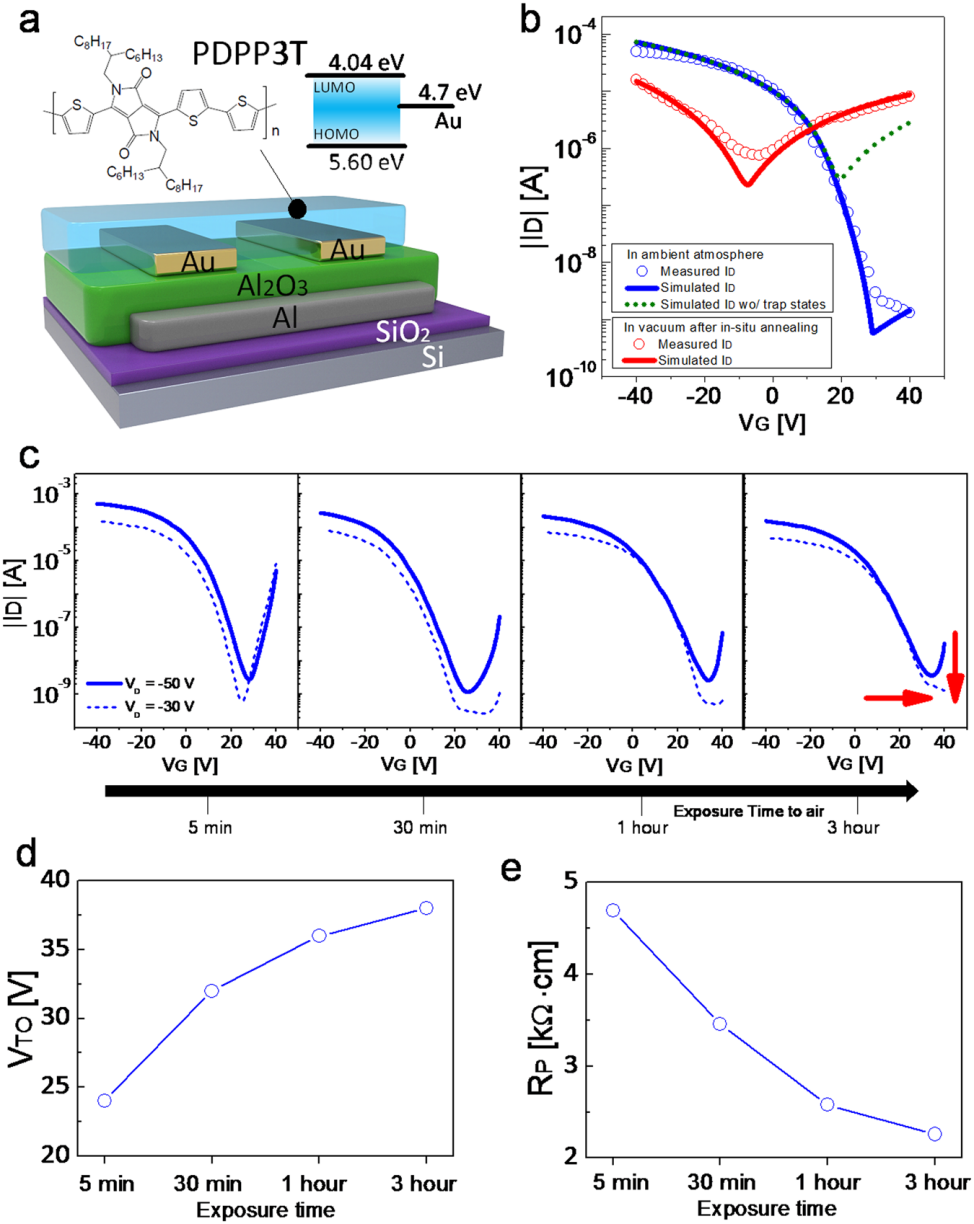
PDPP3T ambipolar transistor architecture and characteristics as a function of air exposure time. (**a**) PDPP3T molecular structure, its schematic and the energy band level. (**b**) Measured (symbols) transfer characteristics of PDPP3T ambipolar transistor at V_D_ = −30 V in ambient atmosphere (blue) and in vacuum after annealing (red). Lines indicate results from 2D numerical simulations. Blue (green) line: simulation of air exposed PDPP3T OTFTs with (without) trap states. Red line: simulation of vacuum annealed PDPP3T OTFTs. (**c**) Measured transfer characteristics at V_D_ = −50 V and V_D_ = −30 V as a function of the air exposure time. (**d**) Turn-on voltage (V_TO_) of PDPP3T transistor as a function of the air exposure time. (**e**) Extracted contact resistance value of PDPP3T transistor as a function of the air exposure time.

First, we fabricate conventional ambipolar OTFTs using PDPP3T, a recently developed DPP based donor-acceptor ambipolar copolymer^23, 53^, as active layer in a bottom-gate bottom-contact geometry. The cross-section of a transistor is shown in Fig. 1a. The channel length and width are L = 6 µm and W = 810 µm, respectively. The details of the fabrication method are given in the *Methods* section. As shown in Fig. 1b, the PDPP3T OTFTs operated in air exhibit unipolar p-type only characteristics (blue circles) and the electron transport is completely suppressed. In contrast, the same OTFTs show ambipolar transfer characteristics (red circles) when measured in vacuum after thermal treatment. The transfer characteristics of vacuum annealed PDPP3T OTFTs as a function of air exposure time are shown in Fig. 1c. As the exposure time is increased, the transfer characteristics shift towards positive gate voltages and the electron conduction is progressively suppressed (Fig. 1c). The transition voltage from p-type to n-type operation, namely the turn-on voltage, is monitored over the exposure time. Fig. 1d shows that the turn-on voltage (V_TO_) shifts from about 0 V to 24 V after 5 minutes of air exposure, and V_TO_ = 38 V after 3 hours. In order to further investigate the effect of air exposure on the hole transport in PDPP3T OTFTs, we extracted the width-normalized contact resistances (R_P_) as a function of the exposure time from the OTFTs electrical characteristics^54^. Fig. 1e shows that the contact resistance decreases from about 5 kΩ·cm to 2 kΩ·cm, which suggests that the hole injection is enhanced.

### Photoelectron spectroscopy

**Figure 2:**
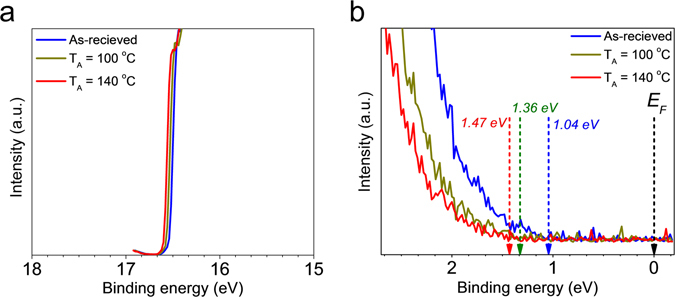
UPS analysis of the PDPP3T. (**a**) Cutoff region. (**b**) Fermi-edge regions. Blue line: as-received. Green line: vacuum annealing at 100 °C. Red line: vacuum annealing at 140 °C.

To investigate the effects of air exposure and thermal treatment, the electronic and physical structures of the PDPP3T films are studied by means of UPS and XPS in an ultra-high vacuum chamber. We measured UPS of PDPP3T films as-received (without annealing), and with *in-situ* thermal treatments, namely annealed at T_A_ = 100 °C and T_A_ = 140 °C. The changes in the electronic properties of PDPP3T film are probed by UPS with He I resonance line (photon energy 21.2 eV). Details on the extraction of the molecular orbital levels from UPS spectra are reported in the *Methods* section. The Fermi energy level E_F_ = −4.6 eV is obtained by the secondary edge cutoff. The change in E_F_ at various temperatures is very small (ΔE_F_ = 0.05–0.06 eV, Fig. 2a). However, Fig. 2b shows that the leading edge of the HOMO with respect to E_F_ gradually moves down from −1.04 eV to −1.47 eV (0.43 eV down-shift) as the temperature is increased by *in-situ* vacuum annealing. The UPS measurements reveal that the HOMO and LUMO levels are down-shifted by *in-situ* vacuum annealing while E_F_ is almost pinned. According with previous works^51, 55–57^, the energy level shift can be attributed to atmospheric dipoles located at the surface of the PDPP3T.

**Figure 3:**
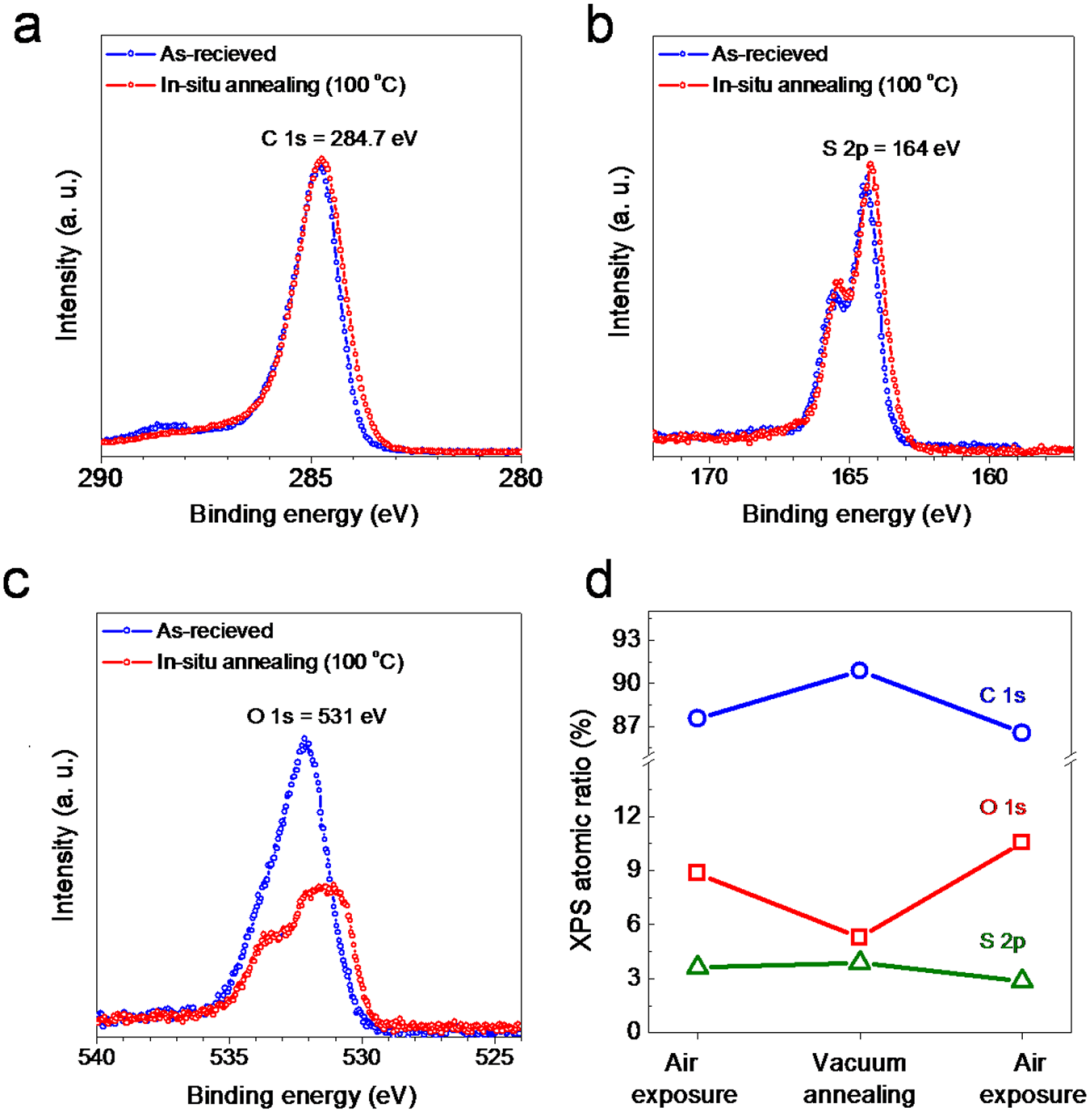
XPS analysis of the PDPP3T. (**a**) C 1s core level spectra. (**b**) S 2p core level spectra. (**c**) O 1s core level spectra. Blue circle: as-received. Red circle: vacuum annealing at 100 °C. (**d**) XPS atomic ratio variations for C 1s, S 2p, and O 1s when the PDPP3T film was measured after the air exposure and the vacuum annealing.

In order to further investigate the effect of vacuum annealing, we perform XPS measurements with *in-situ* thermal treatments and after air exposure. Fig. 3a–c shows XPS spectra for C 1s, S 2p, and O 1s of PDPP3T films. The O 1s spectra are greatly reduced with *in-situ* annealing while C 1s and S 2p spectra do not change. Furthermore, we calculated XPS relative atomic ratios of C 1s, S 2p, and O 1s by integrating peak area values of all species. Fig. 3d shows that the relative concentration of O 1s increases when the PDPP3T is exposed to air and decreases when it is annealed in vacuum. This suggests that the atmospheric oxygen derivatives such as molecule oxygen and water present on the PDPP3T films exposed to air (with the diffusion of the species) are eliminated by the thermal treatment. In addition, we found that the diffused oxygen-related gas molecules existed in the bulk not the surface of film. We conducted XPS with both lower-energy (650 eV) and higher-energy photon source (1486.6 eV) to measure surface region and bulk region, respectively. With 650 eV photon energy, the intensity of O 1s was not changed by the *in-situ* annealing. With 1486.6 eV, on the other hand, we found the intensity of O 1s was reduced after the *in-situ* annealing as shown in Supplementary Fig. 10. This result indicates that annealing is necessary to eliminate the diffused gas molecules in the film. Our bottom-gate bottom-contact device is affected by the diffused oxygen-related gas molecules in the bulk since the charge transport channel is located at the bottom of the film.

### 2D numerical simulations

To gain more insight, we reproduced the measurements of vacuum annealed and air exposed PDPP3T OTFTs by means of 2D numerical simulations (Fig. 1b, full lines). The continuity, Poisson, and drift-diffusion transport equations are solved on a 2D grid. The charge flow at the metal–semiconductor interface is calculated by means of the thermionic field-emission theory, accounting for the actual 2D energy barriers, energy disorder, and the electric field distribution at the contacts^31, 58^. The simulation details are given in the *Method* section and input parameters are given in Supplementary Tables 1 and 2. For 2D numerical simulations, we used the gold work function to be −4.7 eV based on the UPS measurement (Supplementary Fig. 9), which is in good agreement with the −4.5–−5.5 eV energy range^59, 60^. Based on the molecular energy levels from CV measurement (Supplementary Fig. 7) and the results reported by Janssen *et al*.^53^, we used −3.61/−5.17 eV for the LUMO/HOMO level of the PDPP3T device before annealing. For the same device annealed at 140 °C, we used −4.04/−5.60 eV for the LUMO/HOMO level by adding the 0.43 eV down-shift based on the UPS results. Consequently, the charge injection barriers of electrons and holes are estimated to be approximately Φ_Be_ = 0.66 eV and Φ_Bh_ = 0.90 eV. Dipoles due to the ODPA treatment used for reducing the gate leakage current^61–63^, are included by means of surface charges at the insulator-semiconductor interface N_is_ = 2.5 × 10^12^ cm^−2^, which is in agreement with our previous report^32^. The interface charges are readily estimated from the transition voltage, V_TS_ = −5 V of the ambipolar characteristics shown in Fig. 1b (red circles).

**Figure 4:**
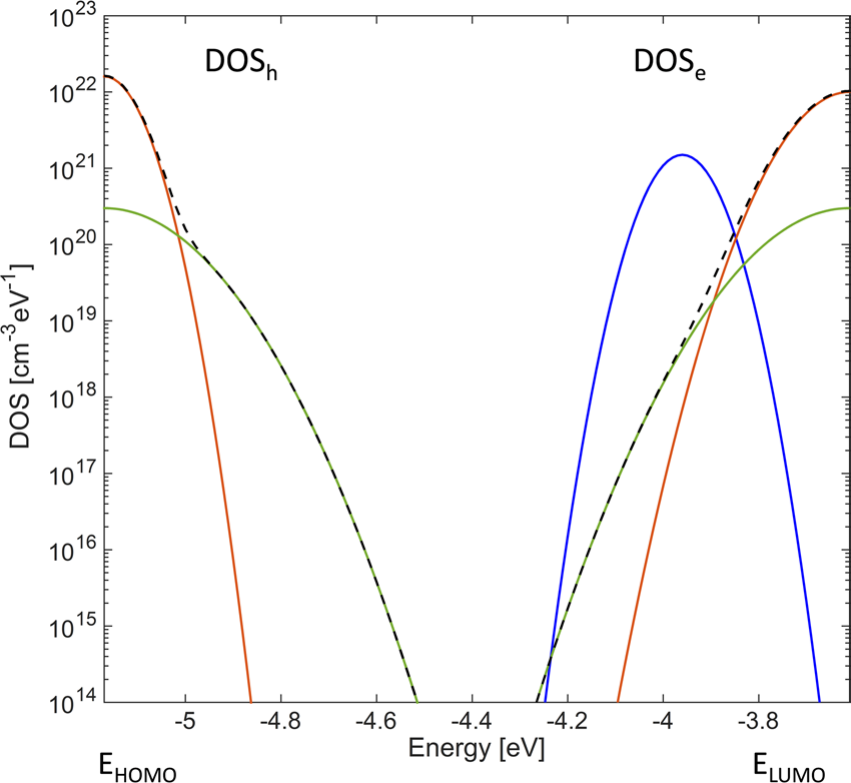
Electron and hole density of states. The dashed line is the overall DOS approximated by the sum of two Gaussian functions. The tail (red line) and deep (green line) states are also shown. The electron DOS parameters are the following. Total density of LUMO tail and deep states are N_te_ = 2 × 10^21^ cm^−3^ and N_de_ = 1 × 10^20^ cm^−3^, respectively. The energy width of the LUMO tail and deep states are σ_te_ = 80 meV and σ_de_ = 120 meV, respectively. The hole DOS parameters are the following. Total density of HOMO tail and deep states are N_th_ = 2 × 10^21^ cm^−3^ and N_dh_ = 1 × 10^20^ cm^−3^, respectively. The energy width of the HOMO tail and deep states are σ_th_ = 60 meV and σ_dh_ = 120 meV, respectively. An additional Gaussian distribution of trap states (full blue line) describes the oxygen-induced. The Gaussian distribution is located at 0.35 eV below the LUMO level, with a total density of states N_tO2_ = 2 × 10^20^ cm^−3^ and a disorder energy width σ_tO2_ = 50 meV. The other simulation parameters are provided in the *Methods* section.

**Figure 5:**
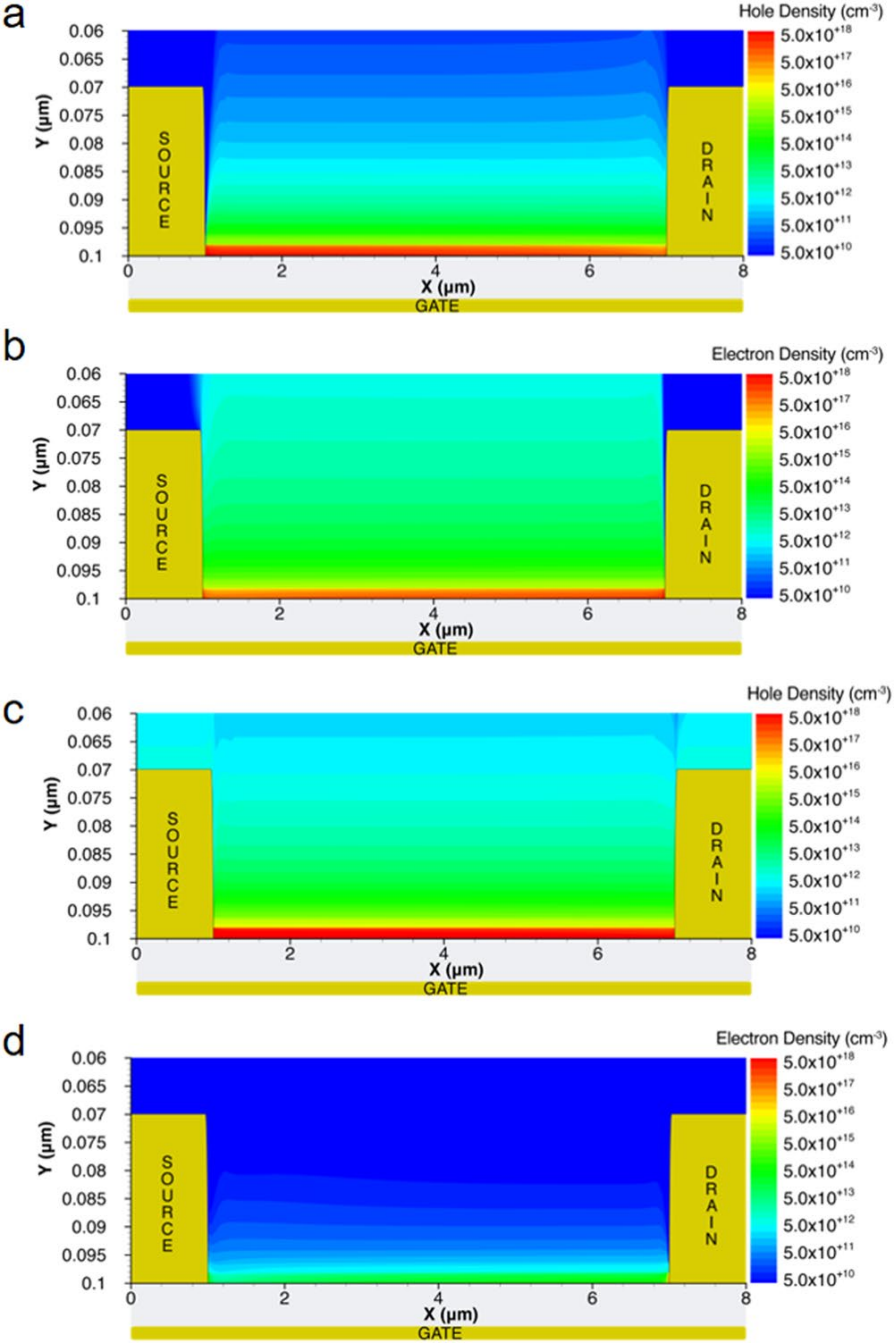
Carrier distribution in PDPP3T ambipolar transistors. 2D numerical simulations. The applied voltages are V_D_ = −30 V, and V_S_ = 0 V. (**a**) P-type operation (V_G_ = −40 V) and (**b**) N-type operation (V_G_ = 40 V) without the atmospheric effects. (**c**) P-type operation (V_G_ = −40 V) and (**d**) N-type operation (V_G_ = 40 V) with the atmospheric effects.

Fig. 4 shows the electron and hole density of states (DOS) obtained from the 2D numerical simulations. In both cases the DOS (dashed black line) can be well approximated by the sum of two Gaussian functions, one for the tail states (full red line) and one for the deep states (full green line). We found that the electron and hole DOS are similar. More in detail, the total number of LUMO and HOMO states are the same (N_te_ = N_th_ = 2 × 10^21^ cm^−3^), while the electron energy disorder (σ_e_ = 80 meV) is slightly larger than the hole energy disorder (σ_h_ = 60 meV). Fig. 5a and b show the calculated hole and electron concentrations inside the vacuum annealed PDPP3T OTFTs. In Fig. 5a V_G_ = −40 V, holes are injected from the source electrode (biased at V_S_ = 0 V) while in Fig. 5b V_G_ = 40 V electrons are injected from the drain electrode (biased at V_D_ = −30 V). In both cases the injected charge carriers are accumulated at the insulator-semiconductor interface, forming the channel. The hole concentration is of about 8 × 10^18^ cm^−3^ and the electron concentration is of about 2 × 10^18^ cm^−3^. The slightly larger hole concentration can be attributed to the larger electron disorder (σ_e_ > σ_h_).

### Analysis of atmospheric effects on PDPP3T semiconductor

Using the UPS and XPS characterization of the PDPP3T semiconductor in combination with the 2D numerical simulations and the electrical characterization, we investigate the effect of air exposure on PDPP3T OTFTs. The UPS characterization reveals that after air exposure the LUMO and HOMO levels of PDPP3T are up-shifted by about 0.43 eV and become −3.61 and −5.17 eV, respectively. Therefore, the charge injection barriers to electrons and holes are estimated to be approximately Φ_Be_ = 1.09 eV and Φ_Bh_ = 0.47 eV. The up-shift of HOMO level enhanced the hole injection because the reduction of Φ_Bh_ in air (=0.47 eV) compared to Φ_Bh_ in vacuum (=0.90 eV) resulted in thinner width of Schottky barrier (SB) at the HOMO-Au junction (Fig. 1e). Moreover, the XPS characterization suggests that the air exposure causes an increase of the oxygen and the water level in the polymer. These two molecules show different nature of interaction. In the presence of both water and oxygen, the PDPP3T polymer anion can be oxidized according to the following chemical equation^51, 64^:

1 $${{\rm{O}}}_{2}+2{{\rm{H}}}_{2}{\rm{O}}+4{{\rm{pol}}}^{-}\rightleftarrows 4{\rm{pol}}+4{{\rm{OH}}}^{-}$$

This, in turn, causes the transfer of electrons from the polymer chain to the OH^−^ groups, where mobile electrons are removed and cannot contribute to transport^48^. In a transistor, this leads to the formation of a matrix of fixed OH^−^ ions in the channel, which causes a positive shift of the turn on voltage, without any polymer degradation (so that the vacuum annealing can recover n-type charge properties). The oxidation potential of the polymer anion can be considered equal to its electron affinity (the lowest unoccupied molecular orbital (LUMO) energy level). As a rule of thumb, it is generally assumed that the higher is the electron affinity of the polymer, the higher is the stability of the polymer anion in air. According to the previous work^51^, a LUMO level deeper than −3.7 eV should be sufficient to guarantee the stability of the polymer in the presence of water, while a LUMO level deeper than −4.9 eV is needed to avoid polymer anion oxidation in the presence of both water and oxygen. In our case the LUMO energy level of the neutral PDPP3T polymer is located at −3.61 eV and hence the effect of both water and oxygen molecules should be accounted for. This is further confirmed by the measured transfer characteristics as a function of the exposure time shown in Fig. 1c. When the exposure time is increased, the amount of oxygen and water in the PDPP3T film increases, and, consequently, the turn on voltage (Fig. 1d) shifts towards positive voltages. Therefore, we included the effect of the fixed OH^−^ ions in our numerical simulations as surface charges located at the insulator-semiconductor interface. We estimated that the surface charges increased by 6 × 10^12^ cm^−2^ after 3 hours of exposure, thus resulting in an overall surface charges N_is_ = 8.5 × 10^12^ cm^−2^.

Fig. 1b shows the current calculated by accounting for the up-shift of the LUMO and HOMO levels by atmospheric effects. It is worthwhile noting the increased N_is_ (dashed green line) does not explain the measured transfer characteristic of air exposed PDPP3T OTFTs. In fact, the calculated current exceeds the measured current by orders of magnitude when V_G_ > 20 V, while they are in good agreement when V_G_ < 20 V. This can be explained as follows. When V_G_ > 20 V, electron becomes the major carrier for the current transport. The electron current decreases significantly because the electronic coupling between the polymer π electrons and the oxygen molecules results in an increase of the electron trapping rate. In contrast, hole current is dominant when V_G_ < 20 V so that increased electron trap density does not affect current level. Similar to what was previously reported for n-type polymer semiconductors^51, 65^ the electronic coupling eventually gives rise to an intra-gap electron trap level, typically located at 0.3–0.5 eV below the LUMO level. For short exposure time the concentration of traps is not sufficient to reduce the electron current (viz. Fig. 1c, t = 5 min), while for longer exposure time the density of trap sites becomes comparable to the charge density in the accumulation layer, and the electron current drops by orders of magnitude (viz. Fig. 1c, t = 180 min). By reproducing the measurements of air exposed PDPP3T OTFTs over the whole range of applied voltages (Fig. 1b, red full line), we found that the density of trap sites can be approximated with a Gaussian distribution located at 0.35 eV below the LUMO level, with a total density of states N_tO2_ = 2 × 10^20^ cm^−3^ and a disorder energy width σ_tO2_ = 50 meV. The calculated trap states is shown in Fig. 4 (full blue line). Fig. 5c and d show the holes and electrons concentration within the air exposed PDPP3T semiconductor, respectively. We found that the hole concentration in the channel exceeds 5 × 10^18^ cm^−3^ (Fig. 5c), while the electron concentration is lower than 5 × 10^15^ cm^−3^ (Fig. 5d) because of the traps. According to several studies^50, 51, 66^, we found that the polymer interaction with water results in an electrochemical oxidation of the polymer, while the interaction with the molecular oxygen gives rise to a formation of localized electron traps below the LUMO energy level. These findings extend to the case of ambipolar semiconductors^34, 67^ and air-induced electron trapping in n-type polymer semiconductors^51^ as reported in previous works, thus revealing that vacuum annealing is crucial to obtain ambipolar charge transport in PDPP3T.

### Control of hole and electron properties in split-gate ambipolar TFTs

**Figure 6:**
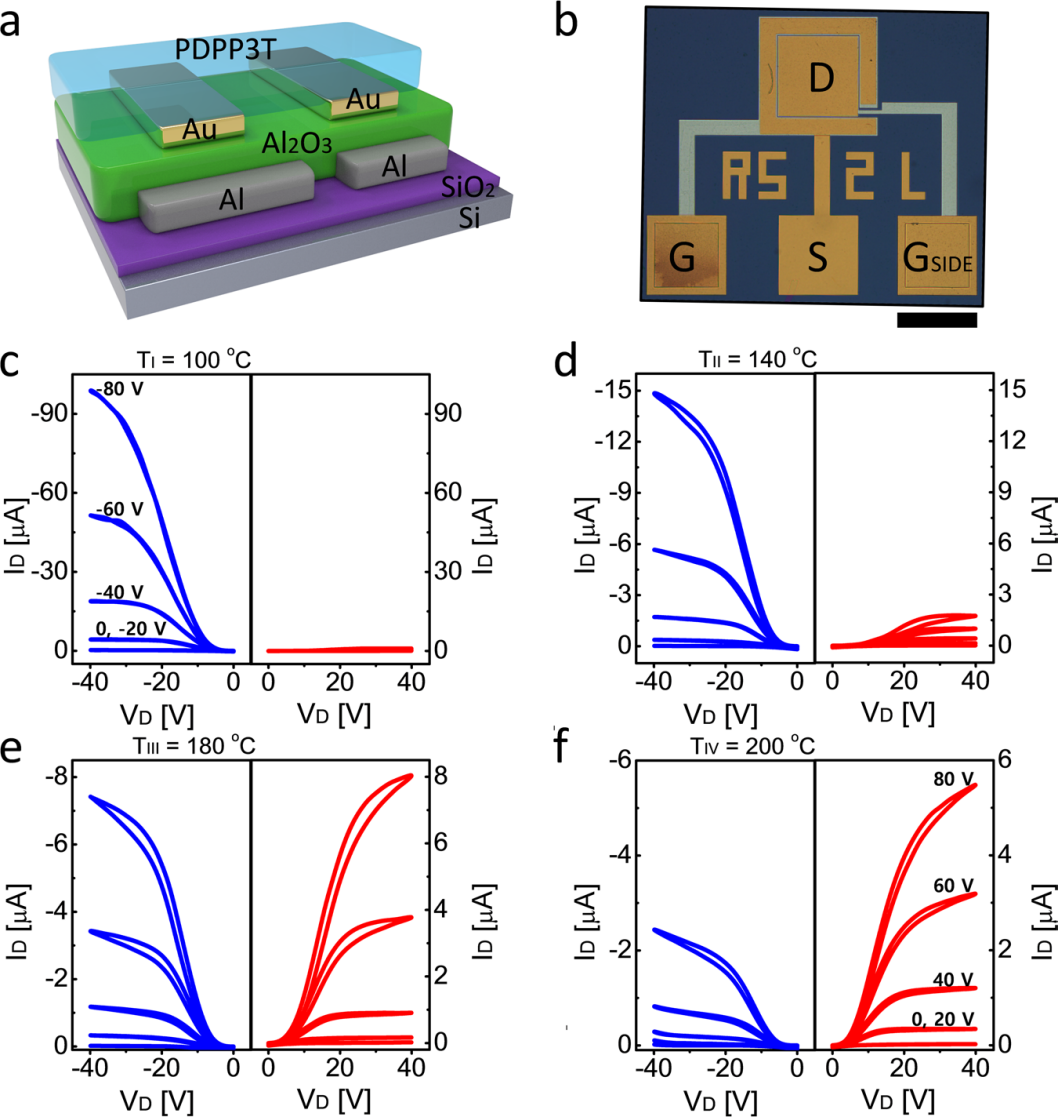
PDPP3T split-gate ambipolar TFTs and their output characteristics as a function of vacuum annealing condition. (**a**) Schematic of device structure. (**b**) Top view optical image. The length of the scale bar is 200 μm (black). Measured output characteristics at (**c**) T_I_ = 100 °C. (**d**) T_II_ = 140 °C. (**e**) T_III_ = 180 °C. (**f**) T_IV_ = 200 °C. Unipolar p-type operation at V_SIDE_ = −60 V. Unipolar n-type operation at V_SIDE_ = 60 V.

In order to investigate the effect of different annealing temperatures on the hole and electron transport in the PDPP3T, we fabricated split-gate OTFTs. It is worth noting that split-gate transistors enable to electrically select the transistor polarity, and, in turn, to easily disentangle and quantify electron and hole conduction. A simplified cross-section and a top view optical image of the split-gate OTFT are shown in Fig. 6a and b, respectively. In a split-gate transistor the gate electrode is divided into two parts, namely the main gate and the side gate. The side gate is used to inhibit the unwanted charge injection at the drain electrode^31, 32^. Four annealing temperatures are applied, thus setting four different conditions: T_I_ = 100 °C (condition I), T_II_ = 140 °C (condition II), T_III_ = 180 °C (condition III), and T_IV_ = 200 °C (condition IV). Thermal treatments are performed in ultra-high vacuum (<10^−6^ torr). Note that all electrical measurements were carried out in room temperature after each annealing treatment. Further details on the thermal treatment conditions and the split-gate ambipolar OTFTs fabrication are given in the *Method* section.

Fig. 6c–f show the measured output characteristics of split-gate PDPP3T OTFTs operating as unipolar p-type (V_SIDE_ = −60 V) or n-type (V_SIDE_ = 60 V) transistors. As the annealing temperature is increased from T_I_ = 100 °C to T_III_ = 180 °C, the hole current decreases while electron current increases. At the optimal annealing condition T_III_, symmetric and balanced p- and n-type output characteristics with almost same drain current (about 8 µA) are obtained. With further increase of the annealing temperature (condition IV, T_IV_ = 200 °C) both electron and hole current were decreased. To investigate the electrical degradation at higher temperature annealing, we conducted further experiments such as atomic force microscopy (AFM), grazing-incidence wide-angle X-ray scattering (GIWAXS), near edge X-ray absorption fine structure (NEXAFS), differential scanning calorimeter (DSC), and thermal gravimetric analysis (TGA). The measurement results revealed that the electrical degradation above 200 °C was not induced by chemical decomposition but by formation of voids inside the semiconductor (Supplementary Figs 1–6 and Table 3).

**Figure 7:**
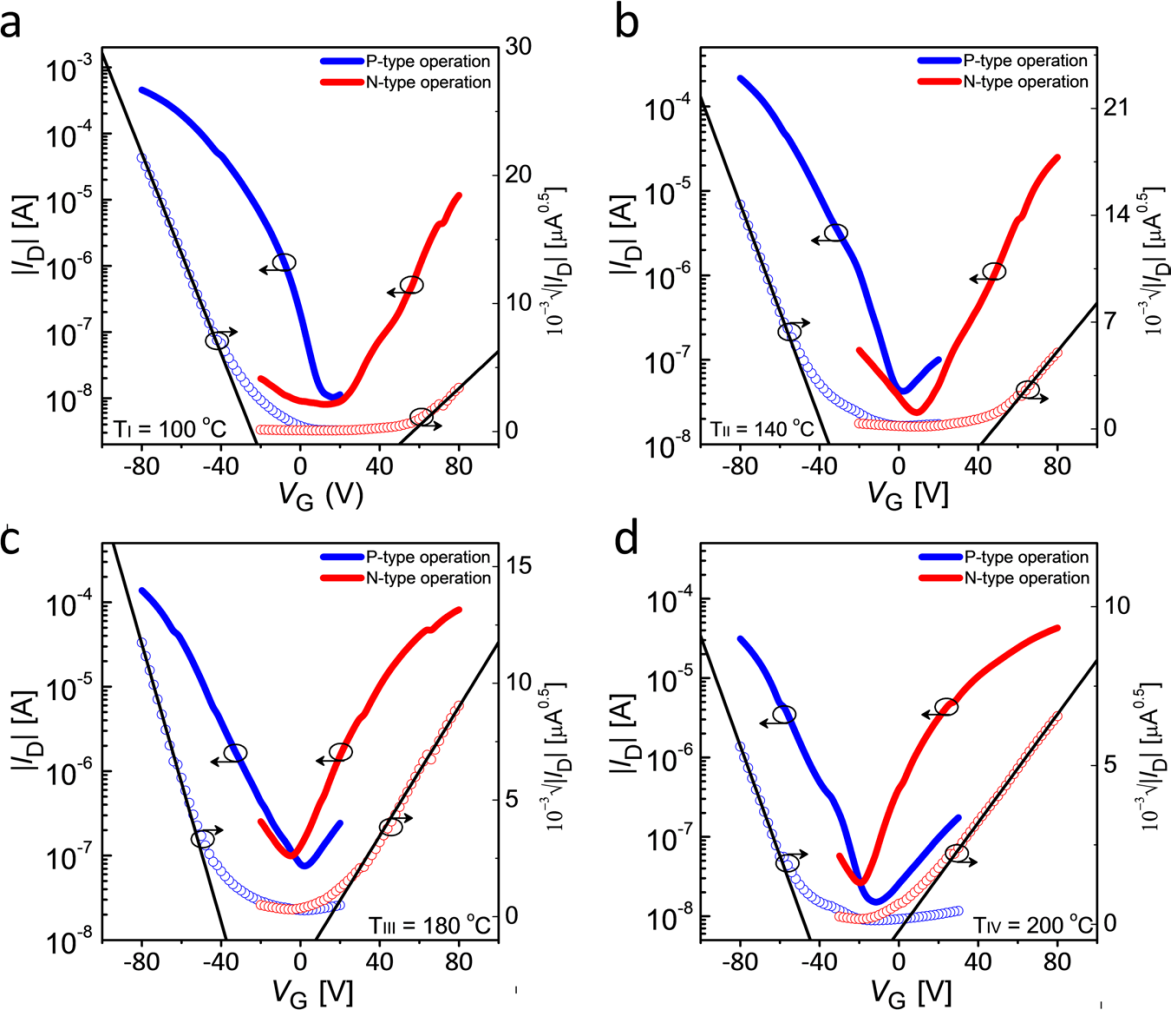
Electrical transfer characteristics of PDPP3T split-gate ambipolar TFTs depending on vacuum annealing condition. (**a**) Measured transfer characteristics at (**a**) T_I_ = 100 °C. (**b**) T_II_ = 140 °C. (**c**) T_III_ = 180 °C. (**d**) T_IV_ = 200 °C. Unipolar p-type operation at V_D_, V_SIDE_ = −60 V. Unipolar n-type operation at V_D,_ V_SIDE_ = 60 V.

**Figure 8:**
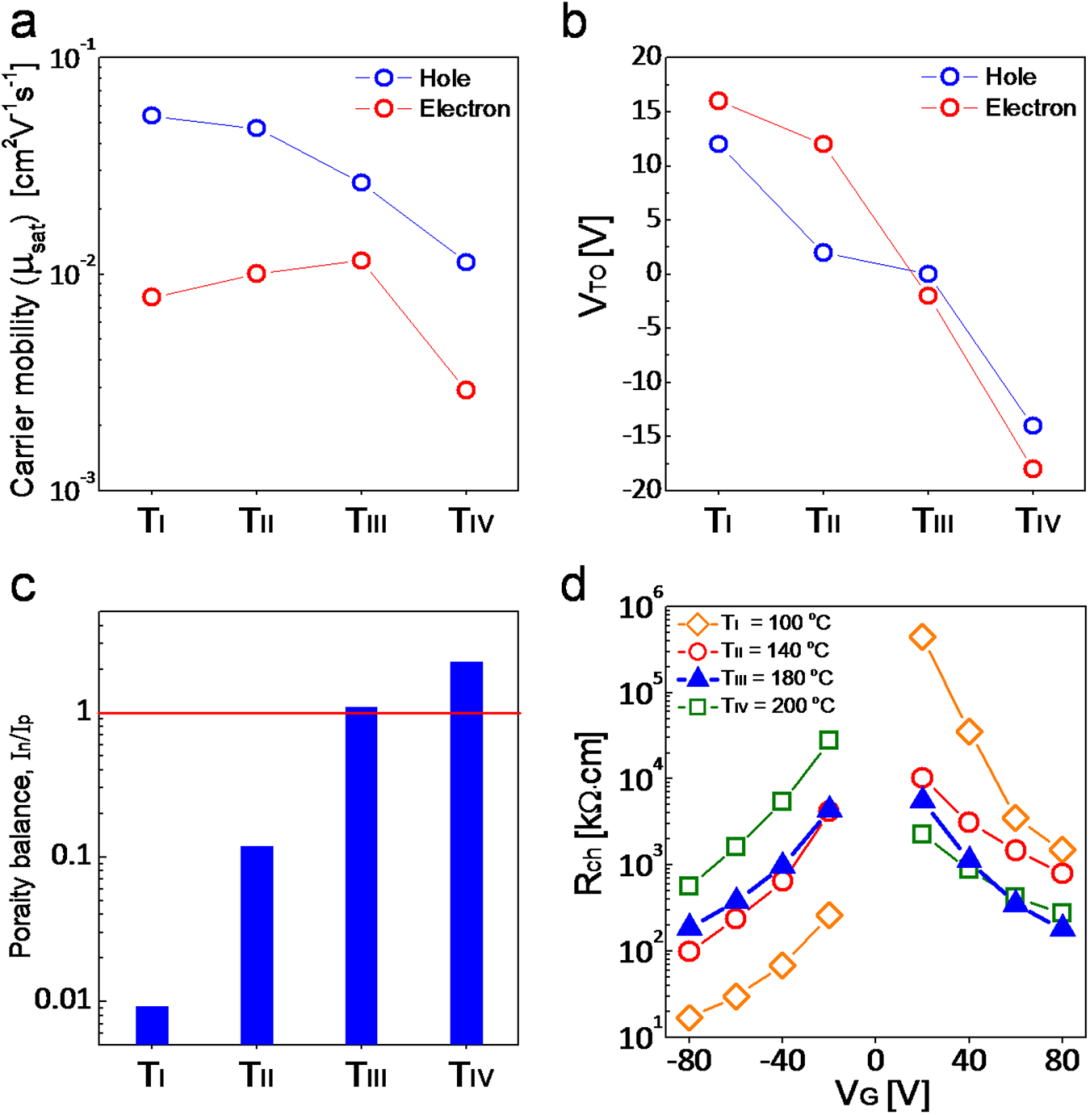
Impact of the vacuum annealing on the transistor electrical properties. (**a**) Extracted saturation mobilities as a function of vacuum annealing condition and (**b**) Turn-on voltage. (**c**) Polarity balance: defined as electron/hole current ratio at |V_G_| = 80 V. |V_D_| = 40 V, and |V_SIDE_| = 60 V. (**d**) Extracted channel resistance of the PDPP3T split-gate ambipolar TFTs. Orange diamond: T_I_ = 100 °C. Red circle: T_II_ = 140 °C. Blue triangle: T_III_ = 180 °C. Green square T_IV_ = 200 °C.

Next, we measured the transfer characteristics of split-gate PDPP3T OTFTs operated as p-type or n-type transistors as a function of the annealing temperature (Fig. 7a–d). We found that at the optimal annealing condition the transfer characteristics are well balanced, with comparable maximum current and turn-on voltage. More in detail, Fig. 8a shows the saturation mobilities in the case of p-type and n-type operations. The mobility for p-type (µ_h_) decreases with the increase of the temperature while the mobility for the n-type (µ_e_) increases until T_III_ = 180 °C and decreases at T_IV_ = 200 °C. At the optimal annealing condition T_III_, we observed similar mobility for both hole and electron conductions, which amounted to µ_h_ = 0.026 cm^2^ V^−1^ s^−1^ and µ_e_ = 0.012 cm^2^ V^−1^ s^−1^, respectively. Fig. 8b shows the turn-on voltage (V_to,p_, V_to,n_) as a function of the annealing temperature obtained when the split-gate PDPP3T OTFT is operated as p-type or n-type transistor. Both V_to,p_ and V_to,n_ are reduced by increasing the temperature and they are comparable at the optimal annealing condition T_III_. Furthermore, we quantified the balancing between hole and electron conduction as the ratio between the corresponding on-currents (I_n_/I_p_) at |V_D_| = 40 V, |V_G_| = 80 V, and |V_SIDE_| = 60 V. As shown in Fig. 8c, the polarity balance are 0.01, 0.12, 1.1 and 2.24 in the cases T_I_ = 100 °C, T_II_ = 140 °C, T_III_ = 180 °C, and T_IV_ = 200 °C, respectively. This confirms that the annealing condition III results in the optimal condition. We further investigate the effect of the annealing temperature by calculating the hole and electron channel resistances (viz. R_hCh_, R_eCh_) from the measured output characteristics^54^. Fig. 8d shows that R_hCh_ increases by raising the annealing temperature while R_eCh_ decreases by increasing the annealing temperature from T_I_ to T_III_. At the optimal annealing temperature T_III_ = 180 °C, R_hCh(III)_ ≈ R_eCh(III)_ in the whole range of gate voltages, thus confirming that balanced hole and electron transport is achieved.

### Complementary inverters

**Figure 9:**
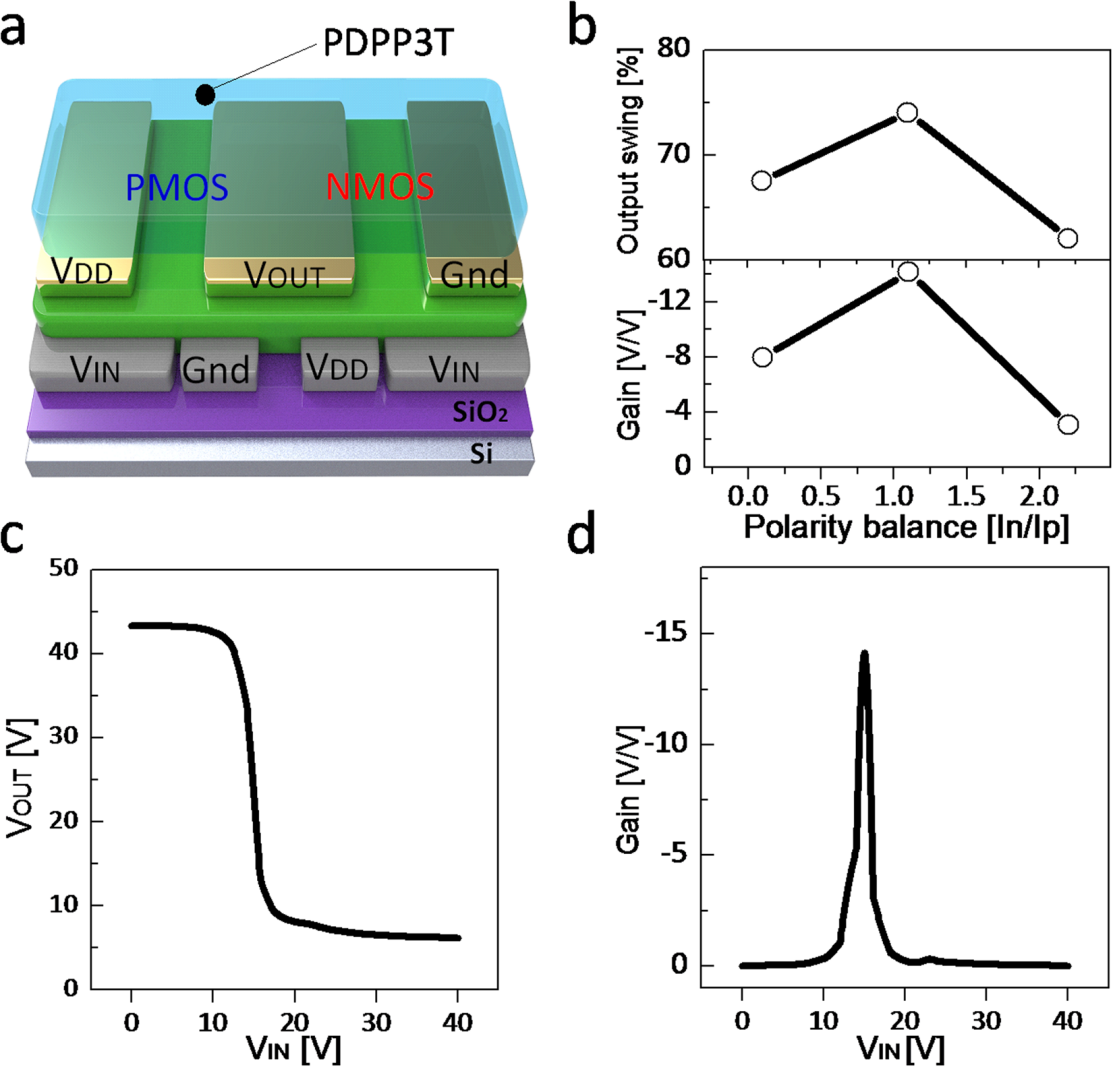
Impact of the polarity balance on the inverter performance. (**a**) Schematic of complementary split-gate PDPP3T inverter structure. (**b**) Output swing and gain as a function of the polarity balance. (**c**) Measured inverter characteristics (V_OUT_ vs. V_IN_) at the optimal condition of T_III_ = 180 °C. (**d**) Gain (=dV_OUT_/dV_IN_) at T_III_ = 180 °C.

As a relevant application example, we fabricated complementary inverters with PDPP3T split-gate OTFTs operated as p-/n-type unipolar transistors. The simplified cross-section of the split-gate complementary inverter is shown in Fig. 9a. In order to evaluate the impact of the annealing temperature on the inverter performance, we measured PDPP3T split-gate inverters at various annealing temperatures (namely T_II_, T_III_, and T_IV_). The ratio between the maximum hole and electron current (I_ONh_/I_ONe_) depends on the thermal treatment and results 1/0.12 (T_II_), 1/1.1 (T_III_), and 1/2.5 (T_IV_). The corresponding inverter transfer characteristics (V_O_-V_I_) are measured and the output swing together with the gain G = dV_O_/dV_I_ are displayed in Fig. 9b. The transfer characteristic obtained at the optimal annealing temperature T_III_ is shown in Fig. 9c and the calculated gain is displayed in Fig. 9d. The maximum gain is larger than 14 (when V_DD_ = 50 V) and 120 (when V_DD_ = 100 V, Supplementary Fig. 14). The output swing is 75% of V_DD_, thus showing superior performances with respect to state-of-art split-gate inverters^31^. In contrast, the inverter performances are seriously degraded when the hole and electron conduction are not well balanced. Indeed, in inverters fabricated with non-balanced ambipolar OTFTs the gain is lower than 5 and the output swing is reduced to 57% of V_DD_.

In summary, we studied the impact of the air exposure and vacuum annealing in the ambipolar PDPP3T OTFTs on balancing the hole and electron conduction. Both conventional coplanar transistors and split-gate transistor architectures have been considered.

Analysis based on UPS, XPS, and 2D numerical simulations reveals that vacuum annealing decreases the hole current while increasing electron current. The combination of the molecular orbitals reorganization and the electron charge trapping by the polymer anion oxidation effect enables to explain the change of hole and electron current characteristics. We observed that hole and electron current of PDPP3T OTFTs were balanced at 180 °C annealing temperature. Furthermore, split-gate transistors showed the symmetric unipolar n- and p-type characteristics with comparable turn-on voltage as well as maximum currents. The detailed study of the effect of air-exposure on electron and hole current in ambipolar OTFTs provides fundamental guidelines for the efficient design of ambipolar polymeric materials and transistors.

We also demonstrated a complementary inverters based on split-gate devices with balanced n- and p-type characteristics. The inverters show the larger gain and output swing than the counterparts with unbalanced n/p characteristics. Therefore, we believe that this work is a step forward towards the development of high-performance complementary ambipolar electronics for low-cost, mass production organic applications.

## Methods

### Photoelectron spectroscopy

Ultraviolet photoelectron spectroscopy (UPS) measurements were performed using He I (hv = 21.2 eV) source from a gas discharge lamp. The energy resolution was better than around 30 meV at room temperature. X-ray photoelectron spectroscopy (XPS) equipped with a photon source (Al-Ka(1486.6 eV) and He I (21.2 eV)) was employed. XPS was conducted at the Korea Basic Science Institute (KBSI).

The cyclic voltammetry (CV) measured value for HOMO level (−5.17 eV) was the same as that in the literature^53^ (Supplementary Fig. 7). Hence, we adopted the HOMO/LUMO level of PDPP3T from the literature for the numerical simulation. The value we took from the UPS measurement of PDPP3T film was the HOMO level down-shift (−0.43 eV) due to vacuum annealing.

### Devices fabrication

Bottom gate/bottom contact conventional and split-gate ambipolar TFTs were fabricated on having SiO_2_/Si substrate. Bottom gate electrodes (aluminum, 200 nm) were deposited using an e-beam evaporator and were patterned using the dry metal etching method. Gate dielectric layers, aluminum oxide (200 nm), were deposited using the atomic layer deposition method. The source/drain electrodes (Au, 100 nm) were deposited on top of the aluminum oxide using e-beam evaporation and lift-off lithography. Inductively Coupled Plasma (ICP) etching was used for oxide etching to form via-holes from bottom electrodes to source/drain electrodes. The samples were dipped in a solution of 10 mM of octadecylphosphonic acid (ODPA) in Isopropyl alcohol (IPA) for 3–5 days after being exposed to UV-ozone for 15 min. Then, PDPP3T, from Solarmer, was dissolved in 1, 2-dichlorobenzene (ODCB) to obtain 13 mg mL^−1^ and spin-coated. The samples were vacuum-annealed at thermal conditions in ultra-high vacuum (<10^−6^ torr) as follows. T_I_ = 100 °C. T_II_ = 140 °C. T_III_ = 180 °C. T_IV_ = 200 °C.

The bottom gate/bottom contact conventional ambipolar TFTs have the channel length and width equal to L = 6 μm and W = 848 μm. The split-gate ambipolar TFTs have the channel length, width, and the gate-gap equal to L = 6 μm, W = 810 μm, and Gap = 1 μm, respectively. All split-gate inverter circuits have a channel length (L) = 12 μm and width (W) = 800 μm for n-type transistor and L = 6 μm and W = 800 μm for p-type transistor.

### Electrical characterizations

All devices were measured in a vacuum probe station (Keithley 4200-SCS) and LCR meter (E4980A). The electrical characterizations of the conventional ambipolar TFTs and the split-gate ambipolar TFTs as a function of the annealing condition were conducted by measuring the same devices, respectively. The saturation mobility was extracted using the following equation (2):

2 $${{\rm{\mu }}}_{\mathrm{sat}}.=\frac{2\cdot {\rm{L}}}{{\rm{W}}\cdot {{\rm{C}}}_{{\rm{ox}}}\,}\cdot {(\frac{\partial \sqrt{{{\rm{I}}}_{{\rm{D}}}}}{{\partial }{{\rm{V}}}_{{\rm{G}}}})}^{2}$$

The measured C_i_ = 40 nF cm^−2^, which is in agreement with a relative permittivity of the Al_2_O_3_ equal to 9. The voltage transfer characteristics (VTC) of the split-gate inverters were measured at V_DD_ = 50 V.

### Two-dimensional numerical simulations

The coupled drift-diffusion, Poisson, and current continuity equations are solved together^30, 32, 58^. The electron and hole DOS are approximated by the sum of two Gaussian functions. An additional Gaussian DOS describes the oxygen-induced trap sites when the OSC is exposed to air. The DOS is shown in Fig. 4 and the DOS parameters are listed in the Supplementary Tables 1 and 2. The simulation parameters are the following: relative permittivity of semiconductor ε_rs_ = 3, relative permittivity of insulator ε_ri_ = 9, hole mobility μ_h_ = 0.1 cm^2^ V^−1^ s^−1^, electron mobility μ_e_ = 0.1 cm^2^ V^−1^ s^−1^, gold electrodes work function Φ_Au_ = −4.7 eV, Schottky barrier lowering ∆Φ_B_ = e [e E/(4 π ε_0_ ε_rs_)]^1/2^, where e is the elementary charge, E is the electric field, and ε_0_ is the vacuum permittivity. The highest occupied molecular orbital (HOMO) energy level of vacuum annealed PDPP3T is E_HOMO_ = −5.60 eV, and the lowest unoccupied molecular orbital (LUMO) energy level is E_LUMO_ = −4.04 eV. After PDPP3T air exposure, E_HOMO_ = −5.17 eV and E_LUMO_ = −3.61 eV.

## Electronic supplementary material

Supplementary Info
