# Supplementary material for: Balancing Hole and Electron Conduction in Ambipolar Split-Gate Thin-Film Transistors
Source: Sci Rep. 2017 Jul 10;7:5015. doi: 10.1038/s41598-017-04933-w (PMC5504072; doi:10.1038/s41598-017-04933-w)
Supplement: Supplementary file 1 — Supplementary Info [file 41598_2017_4933_MOESM1_ESM.pdf]

Supplementary Information

Balancing Hole and Electron Conduction in Ambipolar Split-Gate Thin-Film Transistors

Hocheon Yoo[†1], Matteo Ghittorelli[†2], Dong-Kyu Lee[1], Edsger C. P. Smits[3], Gerwin H. Gelinck[3,5], Hyungju Ahn[4], Han-Koo Lee*[4], Fabrizio Torricelli*[2], and Jae-Joon Kim*[1]

[1] Department of Creative IT Engineering,

Pohang University of Science and Technology (POSTECH), Pohang 790-784, Korea

[2] Department of Information Engineering

University of Brescia, via Branze 38, 25123 Brescia, Italy

[3] Holst Centre, TNO-The Dutch Organization for Applied Scientific Research

High Tech Campus 31, 5656 AE Eindhoven, The Netherlands

[4] Pohang Accelerator Laboratory, Pohang University of Science and Technology (POSTECH)

Pohang 790-784, South Korea

[5] Department of Applied Physics

Eindhoven University of Technology, 5600 MB, Eindhoven, The Netherlands

Table of Contents

Numerical simulation

Supplementary Table 1 & 2. Physical parameters used as input data in the numerical simulations p.S3

Film characterizations of PDPP3T

Supplementary Figure 1. The electrical characteristics of PDPP3T OTFT at various annealing temperatures p.S4

Supplementary Figure 2. AFM images of PDPP3T film at various annealing temperatures p.S5

Supplementary Figure 3,4 & Table 3. GIWAXS analysis of PDPP3T film p.S6

Supplementary Figure 5. NEXAFS analysis of PDPP3T film p.S7

Supplementary Figure 6. DSC and TGA curves of PDPP3T p.S8

Energy level measurement and XPS analysis of PDPP3T

Supplementary Figure 7. CV curves of PDPP3T p.S9

Supplementary Figure 8. UPS analysis of PDPP3T without and with ODPA treatment p.S9

Supplementary Figure 9. UPS analysis of Au p.S10

Supplementary Figure 10. XPS O 1s core level spectra at various photon energy source p.S10

Further device characterizations

Supplementary Figure 11. Electrical characteristics of conventional PDPP3T OTFTs p.S11

Supplementary Figure 12. Current ratio (Ip/In) between the hole and the electron current as a function of bias stress time p.S11

Supplementary Figure 13. Extracted transconductance values as a function of the vacuum annealing condition p.S12

Supplementary Figure 14. DC gain of the split-gate inverter with hole/electron balance (V_{DD} = 80 V, 100 V) p.S12

Highest occupied molecular orbital (HOMO) energy	E_{HOMO} = 5.60 eV
lowest unoccupied molecular orbital (LUMO) energy	E_{LUMO} = 4.04 eV
Total density of HOMO tail states	$N_{th} = 1x10^{21}$ cm^{-3}
Energy width of the HOMO tail-states distribution	σ_{th} = 0.06 eV
Total density of HOMO deep states	$N_{dh} = 1x10^{20}$ cm^{-3}
Energy width of the HOMO deep-states distribution	σ_{dh} = 0.12 eV
Total density of LUMO tail states	$N_{te} = 1x10^{21}$ cm^{-3}
Energy width of the LUMO tail-states distribution	σ_{te} = 0.08 eV
Total density of LUMO deep states	$N_{de} = 1x10^{20}$ cm^{-3}
Energy width of the LUMO deep-states distribution	σ_{de} = 0.12 eV

Supplementary Table 1 | Physical parameters used as input data in the numerical simulations of vacuum annealed PDPP3T.

Highest occupied molecular orbital (HOMO) energy	E_{HOMO} = 5.17 eV
lowest unoccupied molecular orbital (LUMO) energy	E_{LUMO} = 3.61 eV
Total density of HOMO tail states	$N_{th} = 1x10^{21}$ cm^{-3}
Energy width of the HOMO tail-states distribution	σ_{th} = 0.06 eV
Total density of HOMO deep states	$N_{dh} = 1x10^{20}$ cm^{-3}
Energy width of the HOMO deep-states distribution	σ_{dh} = 0.12 eV
Total density of LUMO tail states	$N_{te} = 1x10^{21}$ cm^{-3}
Energy width of the LUMO tail-states distribution	σ_{te} = 0.08 eV
Total density of LUMO deep states	$N_{de} = 1x10^{20}$ cm^{-3}
Energy width of the LUMO deep-states distribution	σ_{de} = 0.12 eV
Total density of the oxygen induced traps states	$N_{tO2} = 2x10^{20}$ cm^{-3}
Energy width of the oxygen induced traps states	σ_{tO2} = 0.05 eV
Central energy of the oxygen induced traps states	E_{O2} = 0.35 eV

Supplementary Table 2 | Physical parameters used as input data in the numerical simulations of air exposed PDPP3T.

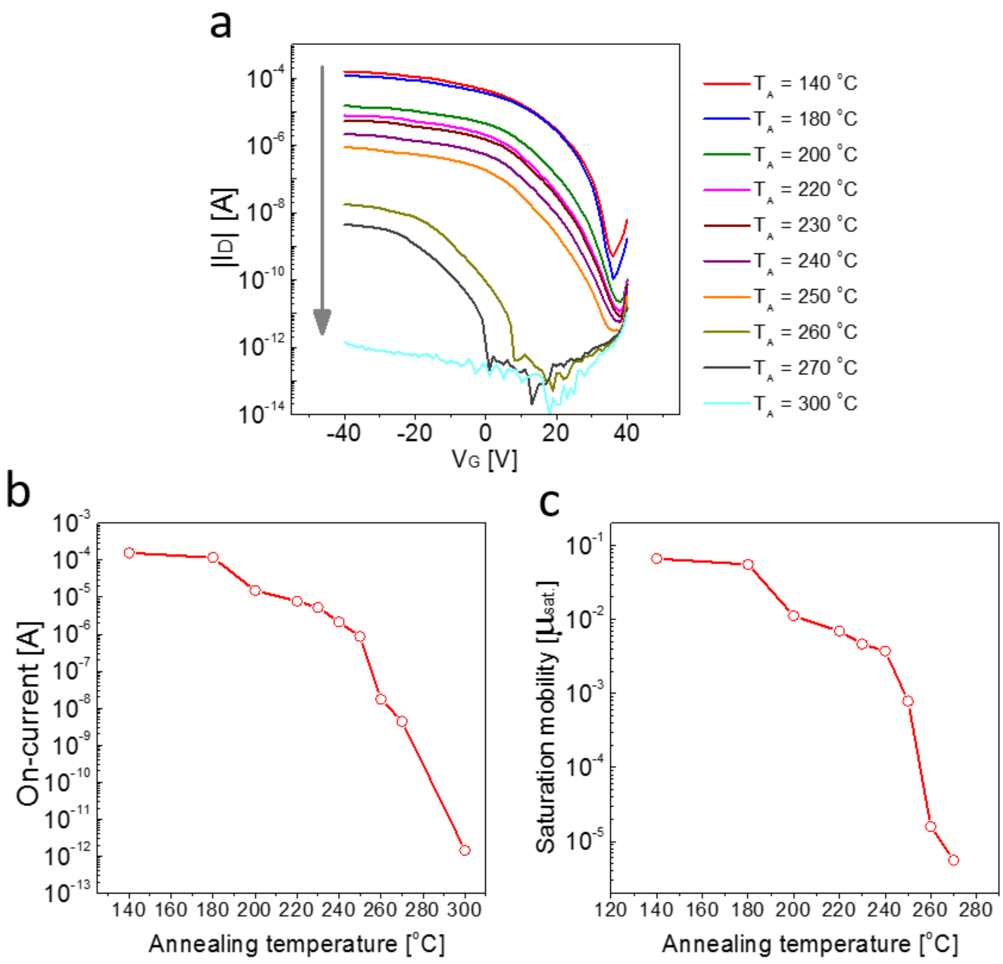

Supplementary Figure 1. (a) I-V curve of PDPP3T OTFT at various annealing temperatures. (b) On-current as a function of annealing temperature. (c) Saturation mobility as a function of the annealing temperature. The measurement was conducted in air to show the electrical degradation by thermal annealing regardless of the degree of air-contamination.

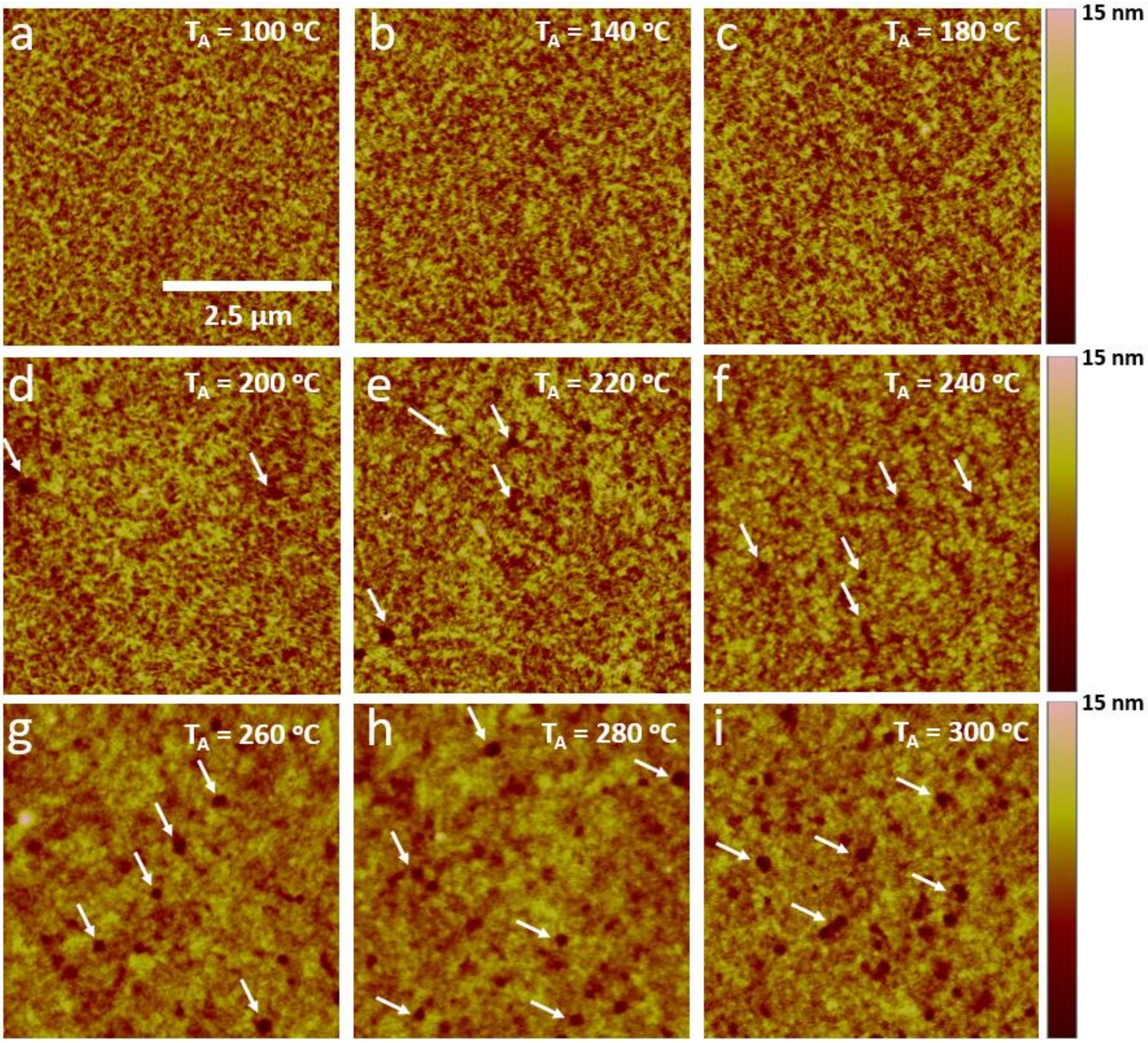

Supplementary Figure 2. Atomic force microscopy (AFM) images of PDPP3T film at various annealing temperatures. (a) T_A = 100 °C. (b) T_A = 140 °C. (c) T_A = 180 °C. (d) T_A = 200 °C. (e) T_A = 220 °C. (f) T_A = 240 °C. (g) T_A = 260 °C. (h) T_A = 280 °C. (j) T_A = 3000 °C. The morphologies of the PDPP3T by the annealing temperature were investigated AFM, Veeco Dimension 3100 and Nanoscope V (Version 7.0)).

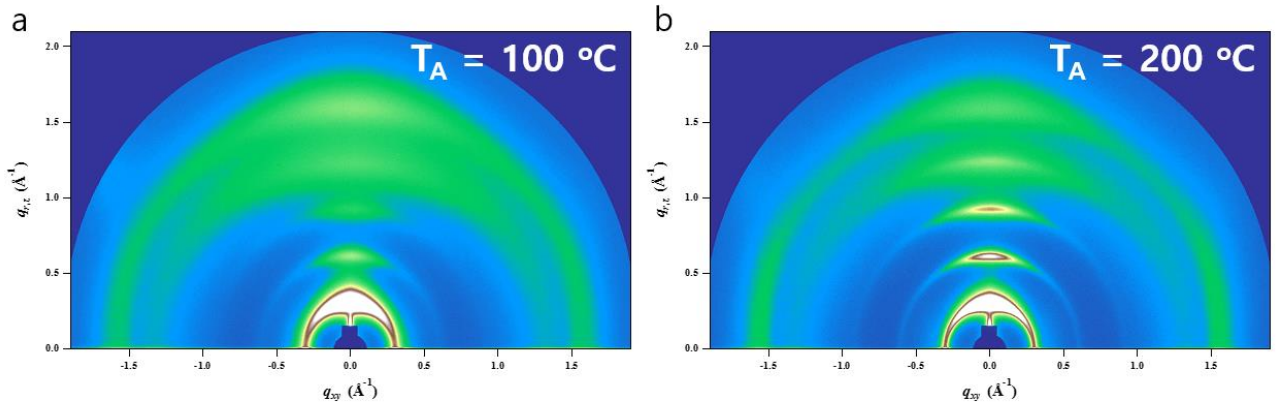

Supplementary Figure 3. Comparison of GIWAXS pattern images between T_A = 100 ºC and T_A = 200 ºC case. 2D-GIWAXS measurement was conducted at 9A beamline of Pohang Accelerator Laboratory (PAL).

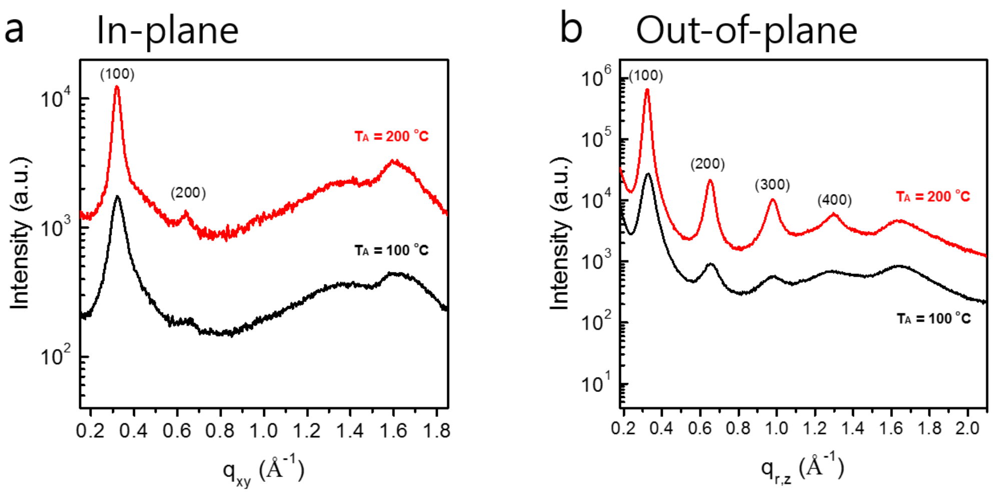

Supplementary Figure 4. Diffraction peak profiles in (a) in-plane (b) out-of-plane.

DPP		$d_{(100)}$ (Å)	$L_{c(100)}$ (Å)	$d_{(\pi-\pi)}$ (Å)
100 ºC	q_{xy}	19.3 Å	69.4 Å	3.81 Å
	q_z	19.2 Å	122.1 Å	3.80 Å
200 ºC	q_{xy}	19.6 Å	96.5 Å	3.85 Å
	q_z	19.5 Å	178.8 Å	3.89 Å

Supplementary Table 3. Structural characteristics of the PDPP3T obtained by GIWAXS.

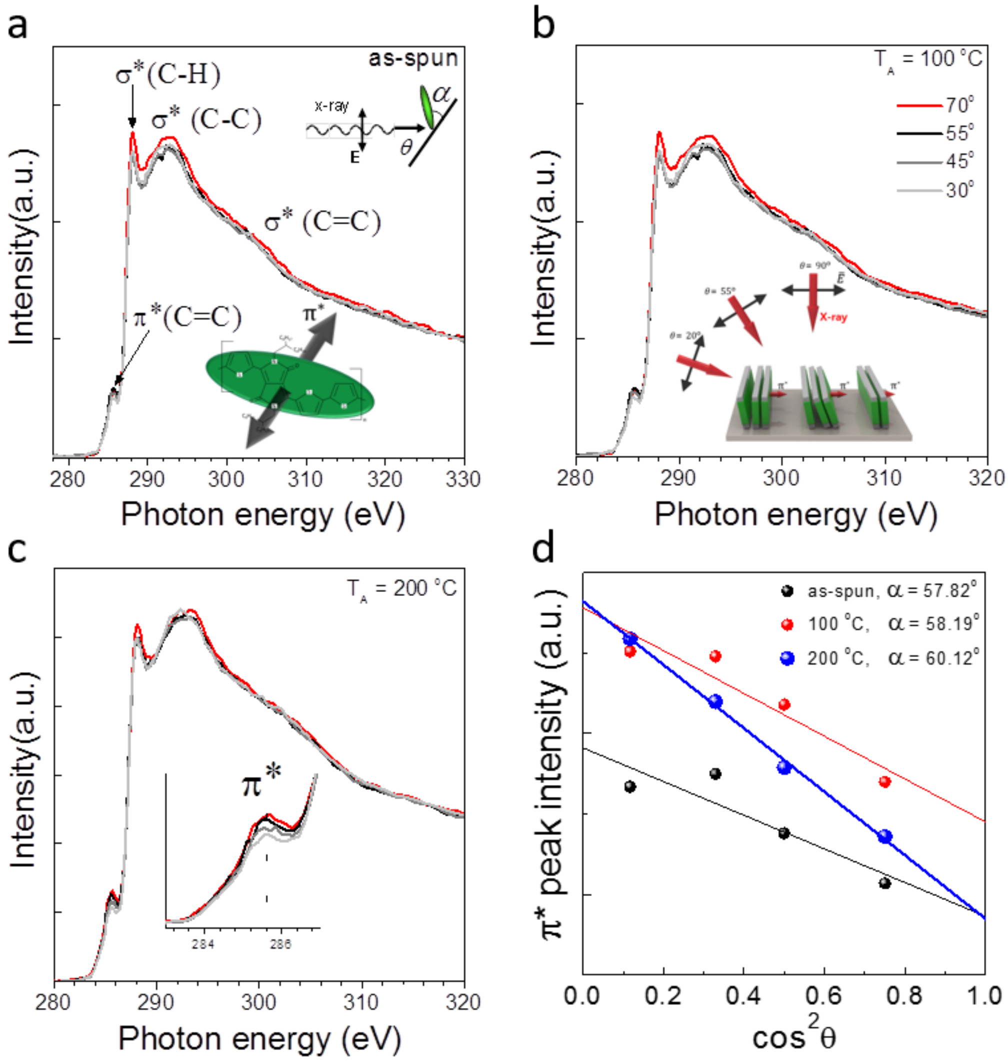

Supplementary Figure 5. NEXAFS results for different annealing temperatures (a) as-spun, (b) 100 ºC, (c) 200 ºC, and (d) Intensities of π* transitions versus incidence angle. NEXAFS measurement was conducted at 4D beamline of Pohang Accelerator Laboratory (PAL).

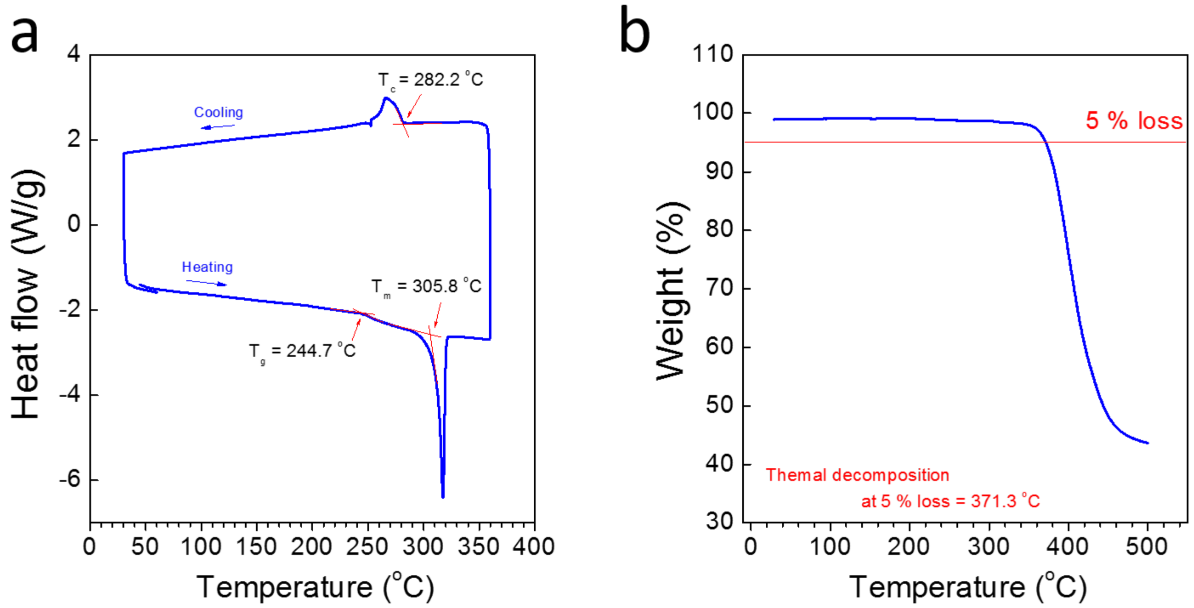

Supplementary Figure 6. Differential scanning calorimetry (DSC) and Thermal gravimetric analysis (TGA) curve of PDPP3T. (a) DSC scans (heating → cooling). Measured glass transition temperature (Tg), crystallization temperature (Tc), and melting temperature (Tm) are 244.7 ºC, 282.2 ºC, and 305.8 ºC, respectively. (b) TGA scan. The thermal decomposition temperature at 5 % weight loss was 371.3 ºC. A heating/cooling rate was 10 ºC/min.

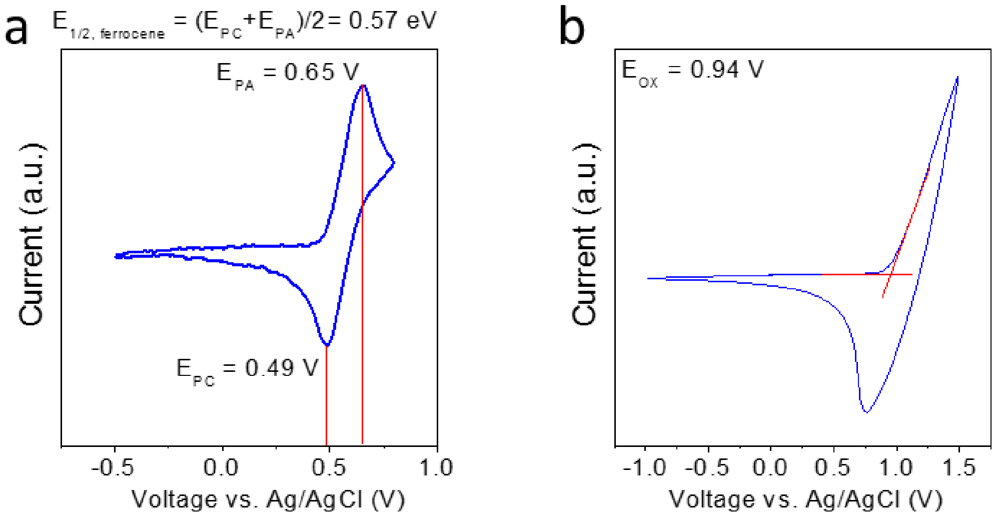

Supplementary Figure 7. Cyclic voltammetry (CV) curve of the PDPP3T. (a) CV curve of the ferrocene/ferrocenuim standard swept in the same condition as that of the compound. (b) CV curve of the PDPP3T in ODCB solution. The measured HOMO value of PDPP3T was -5.17 eV.

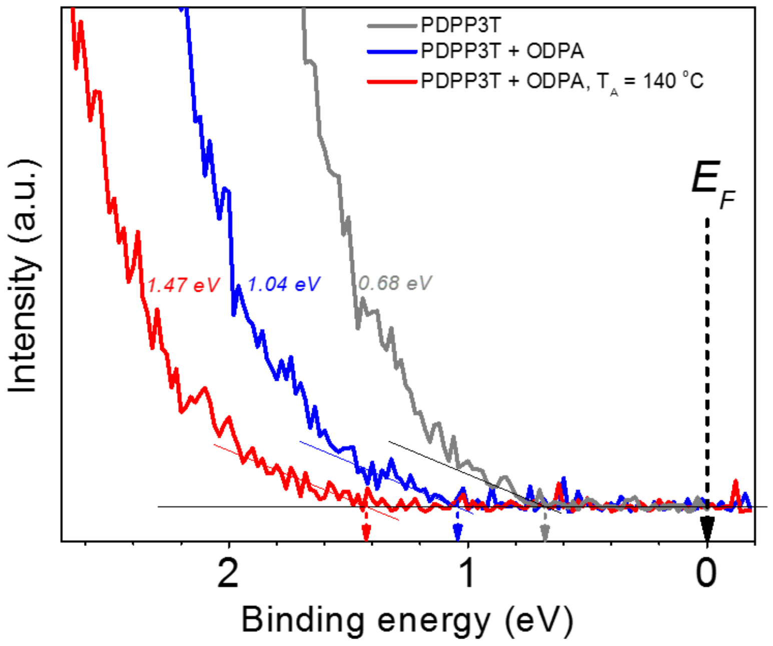

Supplementary Figure 8. UPS analysis of the PDPP3T. Close-up of the HOMO region. Shifts in binding energy are indicated in the figure. Grey line: PDPP3T without ODPA treatment. Blue line: PDPP3T with ODPA treatment. Red line: PDPP3T with ODPA treatment after the in-situ annealing of 140 °C.

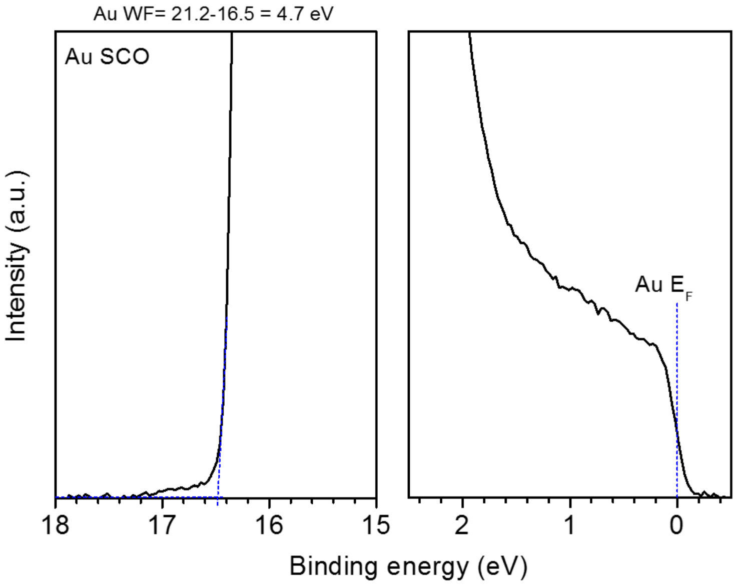

Supplementary Figure 9. UPS analysis of Au. Cut-off binding energy determines the work function of Au.

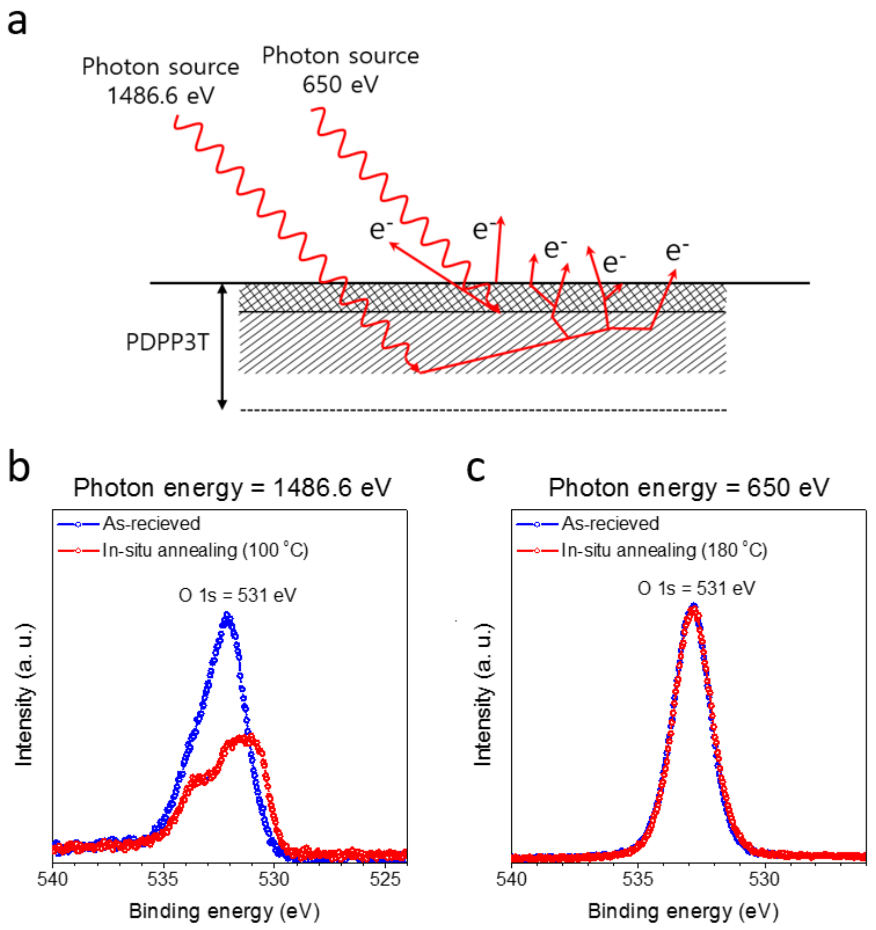

Supplementary Figure 10. (a) Schematic of probing depth of X-ray photon source. (b) O 1s core level spectra when the sample was as-received and in-situ annealing at higher photon energy source (1486.6 eV). (c) O 1s core level spectra when the sample was as-received and in-situ annealing at lower photon energy source (650 eV).

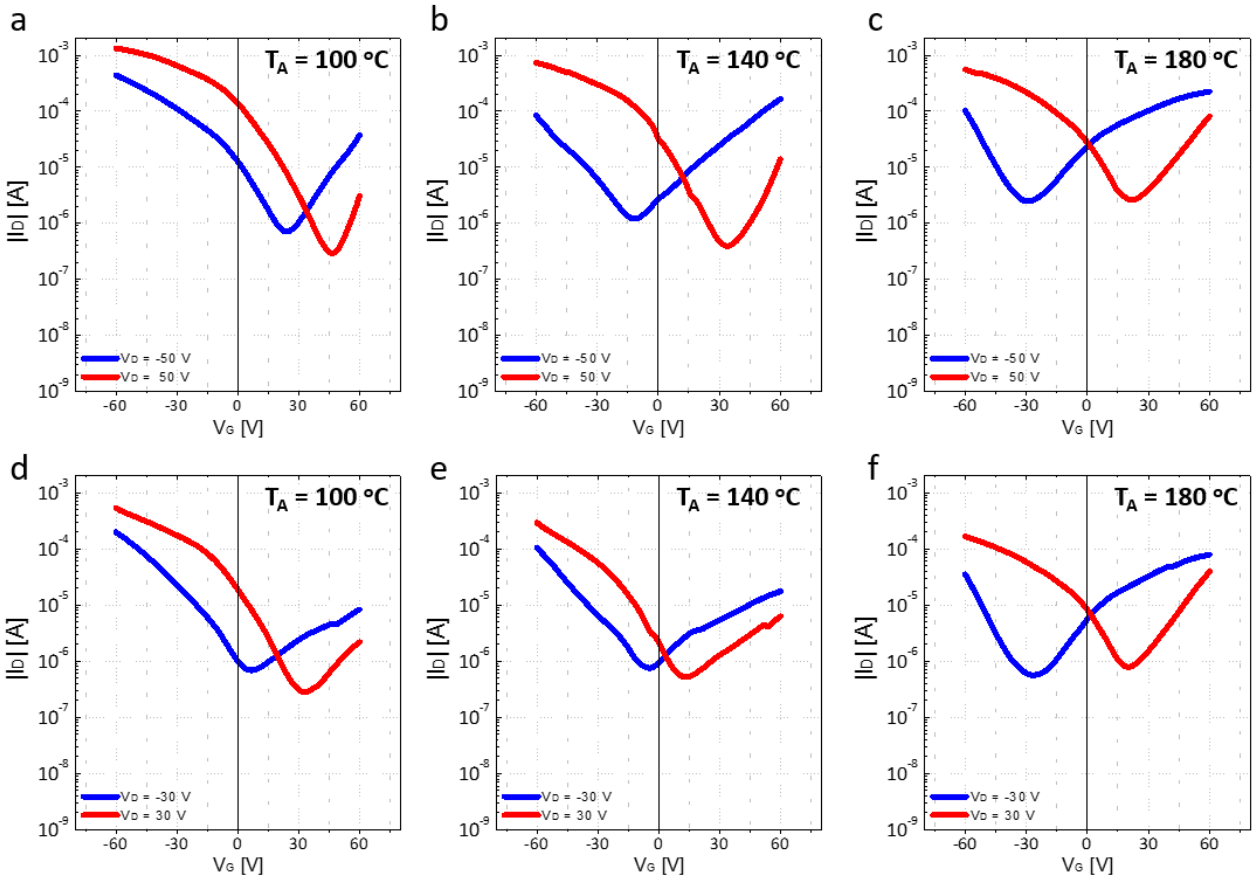

Supplementary Figure 11. IV transfer characteristics of the conventional ambipolar PDPP3T OTFTs. (a) T_A = 100 °C, (b) T_A = 140 °C, and (c) T_A = 180 °C. Blue line: V_D = -50 V. Red line: V_D = 50 V. (d) T_A = 100 °C, (e) T_A = 140 °C, and (f) T_A = 180 °C. Blue line: V_D = -30 V. Red line: V_D = 30 V.

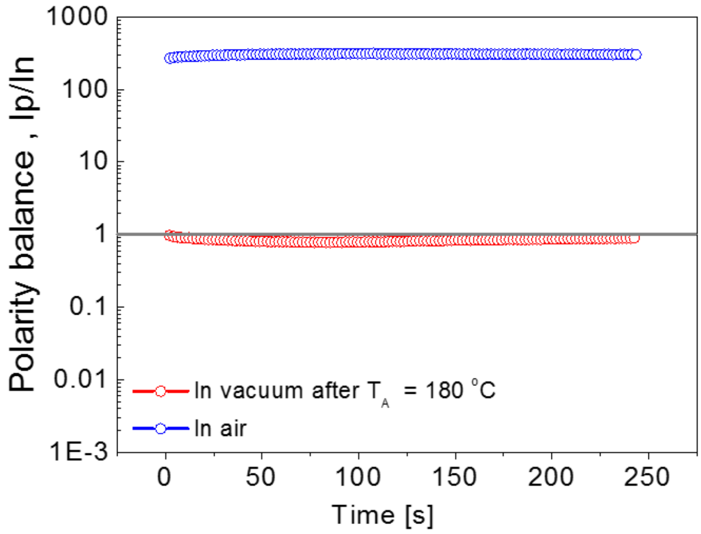

Supplementary Figure 12. Current ratio (I_p/I_n) between the hole and the electron current as a function of bias stress time when the device was measured in vacuum after annealing at T_{III} = 180 °C (red) and in air (blue).

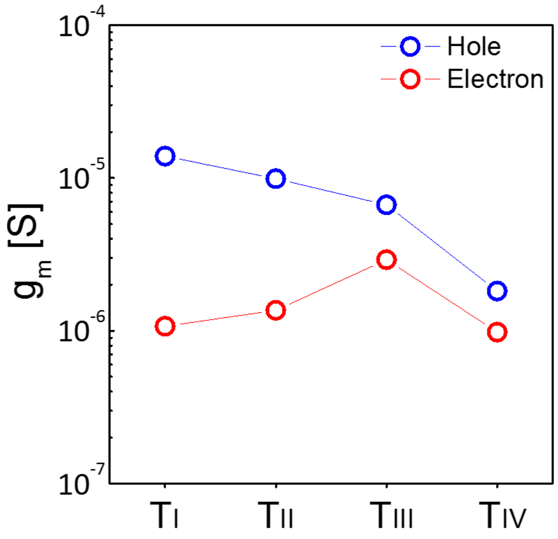

Supplementary Figure 13. Extracted transcnoductance values as a function of the vacuum annealing condition. $|V_D|$ = $|V_{SIDE}|$ = 60 V. T_I = 100 °C. T_{II} = 140 °C. T_{III} = 180 °C. T_{IV} = 200 °C.

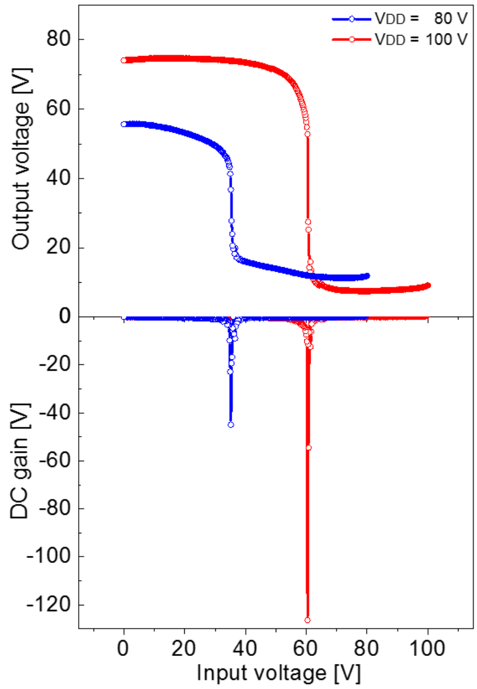

Supplementary Figure 14. The voltage transfer characteristics (VTC) and the corresponding DC gain of the split-gate inverter with hole/electron balance. Blue symbol: V_{DD} = 80 V. Red symbol: V_{DD} = 100 V.
